# MicroRNAs in colorectal cancer: translation of molecular biology into clinical application

**DOI:** 10.1186/1476-4598-8-102

**Published:** 2009-11-14

**Authors:** Ondrej Slaby, Marek Svoboda, Jaroslav Michalek, Rostislav Vyzula

**Affiliations:** 1 Masaryk Memorial Cancer Institute, Department of Comprehensive Cancer Care, Brno, Czech Republic; 2 University Cell Immunotherapy Center, Faculty of Medicine, Masaryk University, Brno, Czech Republic

## Abstract

MicroRNAs (miRNAs) are small non-coding RNAs 18-25 nucleotides in length that downregulate gene expression during various crucial cell processes such as apoptosis, differentiation and development. Changes in the expression profiles of miRNAs have been observed in a variety of human tumors, including colorectal cancer (CRC). Functional studies indicate that miRNAs act as tumor suppressors and oncogenes. These findings significantly extend Vogelstein's model of CRC pathogenesis and have shown great potential for miRNAs as a novel class of therapeutic targets. Several investigations have also described the ability of miRNA expression profiles to predict prognosis and response to selected treatments in CRC patients, and support diagnosis of CRC among cancer of unknown primary site. miRNAs' occurrence has been repeatedly observed also in serum and plasma, and miRNAs as novel minimally invasive biomarkers have indicated reasonable sensitivity for CRC detection and compare favorably with the fecal occult blood test. In this review, we summarize the knowledge regarding miRNAs' functioning in CRC while emphasizing their significance in pathogenetic signaling pathways and their potential to serve as disease biomarkers and novel therapeutic targets.

## Introduction

MicroRNAs (miRNAs) comprise an abundant class of endogenous, small non-coding RNAs 18-25 nucleotides in length that repress protein translation through binding to target mRNAs. The number of verified human miRNAs is still expanding. The latest version of miRBase (release 13.0, March 2009) has annotated over 700 miRNA sequences in the human genome. This number is predicted to double as more miRNAs are awaiting experimental validation. Bioinformatics and cloning studies have estimated that miRNAs may regulate 30% of all human genes and each miRNA can control hundreds of gene targets [1,2]. miRNAs are highly conserved in sequence between distantly related organisms, indicating their participation in essential biological processes. It is well known today that miRNAs have very important regulatory functions in such basic biological processes as development, cellular differentiation, proliferation and apoptosis that affect such major biological systems as stemness, immunity and cancer [3,4].

miRNAs were discovered in the early 1990s by Victor Ambros and colleagues [5]. They found that *lin-4*, a known gene involved in development of the nematode Caenorhabditis elegans, does not code a protein but, instead, gives origin to a small RNA that is 22 nucleotides in length and which was subsequently shown to interact with the 3' untranslated region (UTR) of the *lin-14 *mRNA and to repress its expression [6]. This fascinating form of gene regulation - where a small RNA binds to another RNA - had been largely overlooked for more than 30 years. miRNAs had perhaps escaped detection because of their size as avid gene hunters were mainly interested in long mRNAs and disregarded very short RNAs [7]. Because miRNAs' function had not been clarified, this small molecule RNA was initially considered to be "junk" RNA. Understanding of miRNAs has grown since that early report, and in 2006 Andrew Z. Fire and Craig C. Mello won the Nobel Prize in Physiology or Medicine for their work in understanding of RNA interference and how miRNAs regulate gene expression.

miRNAs have been studied most intensively in the field of oncological research, and emerging evidence suggests that altered miRNA regulation is involved in the pathogenesis of cancers - mainly by regulating the translation of oncogenes and tumor suppressors [1-4]. Findings that miRNAs play a role in cancer biology are further supported by the fact that more than 50% of miRNA genes are located at such chromosomal regions as fragile sites and regions of deletion or amplification that are altered in human cancer [8]. Changes in the expression of miRNAs have been observed in a variety of human tumors. Although expression differences may not necessarily reflect causal events of carcinogenesis, such changes may, nevertheless, regulate genes important in tumor pathogenesis and may be useful for classifying tumors and predicting their outcomes. Such alterations of miRNA expression [1-4] have been detected in the broad spectrum of hematological malignancies and solid tumors, including colorectal cancer (CRC). Here we summarize the recent work on miRNAs, with emphasis on their alterations and roles in CRC pathogenesis and their potential usage as disease biomarkers or novel therapeutic targets.

### MicroRNA biology and function

Many previous studies have revealed abundant knowledge about miRNAs' biogenesis and mechanism of action. Compared with the regulators of gene expression found previously, miRNAs are different in their production and biosynthesis. Early annotation for the genomic position of miRNAs indicated that most miRNAs are located in intergenic regions (> 1 kb away from annotated or predicted genes), although a sizeable minority was found in the intronic regions of known genes in the sense or antisense orientation. This led to the postulation that most miRNA genes are transcribed as autonomous transcription units [1,2]. A detailed analysis of miRNA gene expression showed that miRNA genes can be transcribed from their own promoters and that miRNAs are generated by RNA polymerase II as primary transcripts (pri-miRNAs). These are processed to short 70-nucleotide stem-loop structures known as pre-miRNAs by the ribonuclease called Drosha and the double-stranded-RNA-binding protein known as Pasha (or DGCR8), which together compose a multiprotein complex termed a microprocessor. The pre-miRNAs are transported to cytoplasm by the RAN GTP-dependent transporter exportin 5. In the cytoplasm, the pre-miRNAs are processed to mature miRNAs by their interaction with the endonuclease enzyme Dicer. The resulting 18-25-nucleotide mature miRNA ultimately gets integrated into the RNA-induced silencing complex (RISC) with the central part formed by proteins of the Argonaute family. Mature miRNAs exert their regulatory effects by binding to imperfect complementary sites within the 3' untranslated region (3-UTR) of their mRNA targets, and they repress target-gene expression post-transcriptionally, apparently at the level of translation, through a RISC complex that is similar to, or possibly identical with, that used for the RNAi pathway [3,4]. Consistent with translational control, miRNAs that use this mechanism reduce the protein levels of their target genes, but the mRNA levels of these genes are barely affected. Recent findings indicate, however, that miRNAs sharing only partial complementarity with their targets can also induce mRNA degradation, but it is unclear if translational inhibition precedes destabilization of the gene targets in these cases.

### Involvement of microRNA in colorectal cancer pathogenesis

In general, dysregulation of miRNAs can influence carcinogenesis if their mRNA targets are encoded by tumor suppressor genes or oncogenes. Both, overexpression and silencing or switching off of specific miRNAs, have been described in the carcinogenesis of CRC. Upregulation of mature miRNA may occur owing to transcriptional activation or amplification of the miRNA encoding gene, whereas silencing or reduced expression may result from deletion of a particular chromosomal region, epigenetic silencing, or defects in their biogenesis [9].

**Figure 1:**
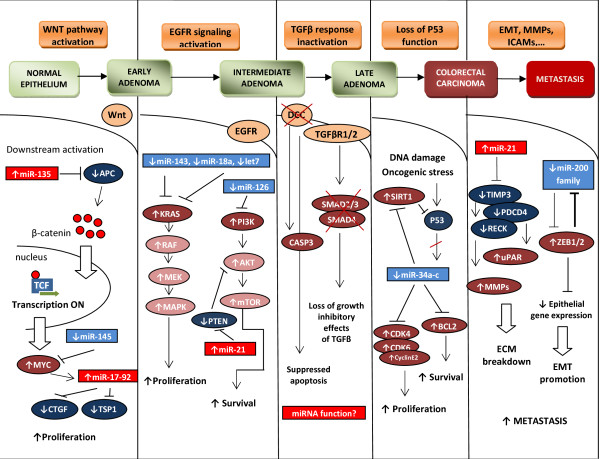
**MicroRNAs' involvement in Vogelstein's model of colorectal cancer pathogenesis**. Particular signaling pathways affected by miRNAs are described in detail in the review (APC - adenomatous polyposis coli, CTGF - connective tissue growth factor, TSP1 -thrombospondin 1, EGFR - epidermal growth factor receptor, mTOR - mechanistic target of rapamycin, PTEN - phosphatase and tensin homolog, DCC - deleted in colorectal carcinoma, TGFβ R1/2 - transforming growth factor, beta receptor 1/2, CASP3 - caspase 3, SIRT1 - sirtuin 1, CDK4,6 - cyclin-dependent kinase 4,6, ECM - extracellular matrix, EMT - epithelial--mesenchymal transition, ICAMs - intercellular adhesive molecules, TIMP3 - tissue inhibitor of metalloproteinase 3, PDCD4 - programmed cell death 4, RECK - reversion-inducing-cysteine-rich protein with kazal motifs, uPAR - plasminogen activator, urokinase receptor, MMPs - matrix metallopeptidases, ZEB1/2 - zinc-finger E-box binding homeobox 1).

Two approaches are applied today to investigate the connection between miRNAs and CRC: functional and profiling studies. On one hand, miRNAs seem to regulate many known oncogenic and tumor suppressor pathways involved in the pathogenesis of CRC. Many proteins involved in key signaling pathways of CRC, such as members of the Wnt/β-catenin and phosphatidylinositol-3-kinase (PI-3-K) pathways, KRAS, p53, extracellular matrix regulators as well as epithelial-mesenchymal transition (EMT) transcription factors [10], are altered and seem to be affected by miRNA regulation in CRC (summarized in Fig. 1). Analyses of these miRNAs in mechanistic studies are crucial to better understanding CRC pathogenesis and with an aim to eventually identify novel therapeutic targets [11,12]. Findings from this area are discussed in the first part of this review. On the other hand, expression profiles of hundreds of miRNAs have been shown to have at least the same potential for identification of biomarkers as profiling of their mRNA or protein counterparts. This allows predicting prognosis and therapy response as well as distinguishing certain disease entities, including CRC, as discussed in the second part of the article that focuses on miRNA expression profiling.

#### Wnt/β-catenin pathway

The Wnt/β-catenin pathway plays a central role in an early colorectal tumor development. Inactivation of the adenomatous polyposis coli (APC) gene is a major initiating event in colorectal carcinogenesis occurring in more than 60% of colorectal adenomas and carcinomas and leading to stimulation of the Wnt pathway via free β-catenin [10]. According to a recent study by Nagel *et al*. [13], miRNAs represent a novel mechanism for APC regulation in CRC. *miR-135a *and *miR-135b *decrease translation of the APC transcript *in vitro*. Of note, *miR-135a *and *miR-135b *were also found to be upregulated *in vivo *in colorectal adenomas and carcinomas and correlated with low APC levels [13]. These observations suggest that alteration in the *mir-135 *family can be one of the early events in CRC's molecular pathogenesis.

#### EGFR signaling (KRAS and phosphatidylinositol-3-kinase pathways)

The epidermal growth factor receptor (EGFR) pathway contribute to promotion and progression of broad spectrum of solid tumors and it is a promising target for anticancer therapy. Stimulation of the EGFR and, subsequently, KRAS signaling lead to the activation of numerous signal transduction molecules initiating a cascade of downstream effectors that mediate tumor growth, survival, angiogenesis and metastasis [14]. KRAS oncogene has been reported to be a direct target of the *let-7 *miRNA family [15]. When *let-7 *low-expressing DLD-1 colon cancer cells were transfected with *let-7a-1 *precursor, significant growth suppression with concurrent decrease of the KRAS protein levels was observed while the levels of both of their mRNAs remained almost unchanged [16]. Another miRNA associated with KRAS regulation in CRC is *miR-143 *[17]. KRAS was deduced to be a *miR-143 *target not only by computational prediction but also by noting the inverse correlation between *miR-143 *and KRAS protein level in clinical samples. KRAS expression *in vitro *was significantly abolished by treatment with *miR-143 *precursor, whereas *miR-143 *inhibitor increased the KRAS protein level. Inhibition of KRAS expression by *miR-143 *blocked constitutive phosphorylation of MAPK [17]. Recently, *miR-18a *was observed to directly regulate KRAS but not N- and HRAS levels in the colon adenocarcinoma HT-29 cells [18].

Another central signaling pathway downstream from EGFR and important in CRC development is the phosphatidylinositol-3-kinase (PI-3-K) pathway. Studies based on microRNA arrays found a ubiquitous loss of *miR-126 *expression in colon cancer lines when compared to normal human colon epithelia and reconstitution of *miR-126 *resulted in a significant growth reduction [19]. The p85β regulatory subunit involved in stabilizing and propagating the PI-3-K signal was mechanistically proven to be a direct target of *miR-126*. Furthermore, this p85β reduction mediated by *miR-126 *was accompanied by a substantial reduction in phosphorylated AKT levels in the cancer cells, suggesting an impairment in PI-3-K signaling. In a panel of matched normal colon and primary colon tumors, each of the tumors demonstrated *miR-126 *downregulation together with an increase in the p85β protein level [19]. Another important regulatory component of the PI-3-K pathway, the tumor suppressor gene PTEN, is strongly repressed by *miR-21*, which was demonstrated on a hepatocellular carcinoma model [20]. However, *miR-21 *is the miRNA most frequently upregulated in CRC [21-23]. It seems that suppression of PTEN controlled by *miR-21 *is associated with augmentation of PI-3-K signaling and progression of CRC.

#### p53 pathway

A well-known tumor suppressor gene, p53 is mutated in about 50-75% of all CRCs and many other human tumors. p53 responds to DNA damage or deregulation of mitogenic oncogenes through the induction of cell cycle checkpoints, apoptosis, or cellular senescence [10]. Although p53 is clearly a transcriptional activator, numerous reports have indicated that p53 also represses the expression of specific genes either directly or indirectly [24]. The manner in which this occurred was obscure, with both transcriptional and post-transcriptional suppression as possible mechanisms. In the latter case, the discovery of an extensive regulatory network of miRNAs offered the possibility that p53-mediated control of miRNA expression could allow it to act indirectly to repress target gene expression at the post-transcriptional level. Recently, several groups have unraveled important aspects of the connection between p53 and the miRNA network [25]. The conserved *miR-34a-c *family was found to be direct transcriptional targets of p53. miRNA expression patterns were analyzed in wild-type p53+/+ and p53-/- mutant HCT-116 colon cancer cell lines after treatment with DNA damaging agents. Several miRNAs were induced in the wild type but not in the p53-/- mutant cells, thus suggesting a p53-mediated expression. *miR-34a *showed the strongest induction. Expression of miR-34a was sufficient to induce apoptosis through p53-dependent and independent mechanisms. miR-34a-responsive genes are highly enriched for those that regulate cell-cycle progression, cellular proliferation, apoptosis, DNA repair and angiogenesis[26]. By experimentally overexpressing *miR-34a*, p53 effects like cell-cycle arrest and apoptosis could be achieved. p53's connection to the *miR-34 *family was successfully evaluated also on a model of lung carcinoma cells harboring regulated p53 alleles [27] and p53+/+ and p53-/- mouse embryo fibroblasts [28]. miR-34a induction leads to dramatic reprogramming of gene expression. Among the downregulated targets of the *miR-34 *family were well-characterized p53 targets like CDK4/6, cyclin E2, E2F5, BIRC3 and Bcl-2. Notably, these effects were nearly identical irrespective of whether *miR-34-a*, *miR-34-b *or *miR-34-c *was introduced [26-28]. Others have identified SIRT1, a member of a highly conserved gene family (sirtuins) encoding NAD(+)-dependent deacetylases and a negative regulator of apoptosis in response to cellular stress, as an additional target of *miR-34a *[29]. Interestingly, the suppression of SIRT1 by *miR-34a *resulted in apoptosis in wild-type colon cancer cells but not in p53-/- mutants. This suggests a positive feedback loop between p53 and *miR-34 *[29].

Decreased levels of the *miR-34 *family have been found in many tumors, including CRC [24]. *miR-34a *expression was found to be downregulated in 9 of 25 CRCs [30]. Loss of 1p36, the genomic interval harboring miR-34a, is common in diverse human cancers [26] but one of the other mechanisms responsible for decrease of *miR-34 *family expression levels seems to be CpG island hypermethylation. *miR-34a *promoter methylation was reported in 3 of 23 cases of colon cancer [31]. *miR-34b/c *were found to be epigenetically silenced in 9 of 9 cell lines examined and in 101 of 111 primary CRC tumors, but they were not in normal colonic epithelium. After treatment with demethylating agents, *miR-34b/c *expression was restored. That resulted in inhibition of tumor motility and metastasis formation [32]. The high frequency of their methylation in CRC and their contribution to the p53 network imply that *miR-34a-c *function as important tumor suppressors which can be lost during CRC development [11].

Like p53, members of the *miR-34 *family can be considered as tumor suppressors to date, making them potential candidates for causing cancer by way of their inactivation.

#### Extracellular matrix breakdown and epithelial-mesenchymal transition

The extracellular matrix (ECM) and its remodeling plays a crucial role in the development of blood supply and interaction with the mesenchymal stroma upon which tumor cells grow. ECM remodeling is one of the necessary conditions of tumor growth, survival, invasiveness, and metastasizing. The key enzymes, among the many involved in ECM breakdown, are proteinases, and among these are the urokinase plasminogen activator (uPA) cascade and the matrix metalloproteinases (MMPs) [33]. Substantial data indicate that miR-21 is significantly elevated in CRC and in many other tumors of various origins [21]. Based upon a glioblastoma model, it was described that *miR-21 *regulates multiple genes associated with cellular motility and ECM remodeling. These included the RECK and TIMP3 genes which are suppressors of malignancy and inhibitors of MMPs [34]. Specific inhibition of *miR-21 *with antisense oligonucleotides leads to elevated levels of RECK and TIMP3 and therefore reduces MMP activities. Although these observations originate from a glioblastoma model, upregulation of *miR-21 *in CRC cells has been shown to increase their migratory and invasive abilities and *miR-21 *seems to act, in this case, in a similar manner [34]. Furthermore, *miR-21 *was shown to act on PDCD4, a tumor suppressor gene that is an independent prognostic factor in resected CRC and a new negative regulator of intravasation through the invasion-related urokinase receptor gene (uPAR) [35]. Silencing of *miR-21 *by *anti-miR-21 *resulted in increased levels of PDCD4 in colorectal cell lines and decreased invasion in a chicken-embryo-metastasis assay. In addition, 22 resected human tumors showed higher *miR-21 *expression than did the corresponding normal mucosa and decreased amounts of PDCD4 protein while mRNA levels were unchanged. These results argue for *miR-21's *having an important function in the pathogenesis of CRC, as it also shows an inverse correlation with survival [35].

Epithelial-mesenchymal transition (EMT) is the conversion of an epithelial cell into a mesenchymal cell. Morphologically, EMT is characterized by a decrease of E-cadherin, loss of cell adhesion, and increased cell motility leading to promotion of metastatic behavior of cancer cells (including CRC) [36]. The transcriptional repressor zinc-finger E-box binding homeobox 1 (ZEB1) is a crucial inducer of EMT in various human tumors, and it recently was shown to promote invasion and metastasis of tumor cells. The functional links to EMT comes from members of the *miR-200 *family (*miR-200a, miR-200b, miR-200c, miR-141 and miR-429*). ZEB1 directly suppresses transcription of *miRNA-200 *family members *miR-141 *and *miR-200c*, which strongly activate epithelial differentiation in pancreatic, colorectal and breast cancer cells [37]. Notably, the EMT activators transforming growth factor β2 and ZEB1 are the predominant targets downregulated by these miRNAs. These results indicate that ZEB1 triggers an miRNA-mediated feedforward loop that stabilizes EMT and promotes the invasion of cancer cells. Alternatively, depending on the environmental trigger, this loop might switch and induce epithelial differentiation, thereby explaining strong intratumoral heterogeneity. A recent study associated the expression of *let-7 *with two differentiation stages of a panel of cell lines (with epithelial and a mesenchymal gene signatures) and linked *let-7 *to EMT [38].

#### MicroRNAs regulating other signaling pathways

Insulin receptor substrate-1 (IRS-1) plays an important role in cell growth and cell proliferation. IRS-1, especially when activated by the type 1 insulin-like growth factor receptor (IGF-IR), sends an unambiguous mitogenic, anti-apoptotic, and anti-differentiation signal. IRS-1 levels are often increased in cases of human cancer and are low or even absent in differentiating cells [39]. *miR-145 *has been proposed as a tumor suppressor and it had been shown previously that *miR-145 *targets the 3' UTR of IRS-1 and dramatically inhibits the growth of colon cancer cells [40]. More recently, IGF-IR was proven to be another direct target of *miR-145*. It was shown that an IRS-1 lacking its 3' UTR is no longer downregulated by *miR-145 *and rescues colon cancer cells from growth inhibition induced by *miR-145*. An IGF-IR resistant to *miR-145 *(again by elimination of its 3' UTR) was not downregulated by *miR-145 *but failed to rescue colon cancer cells from growth inhibition. These data indicate that *miR-145 *plays a significant role in IGF signaling in cancer pathogenesis [41].

The *miR-17-92 *cluster encodes six miRNAs *(miR-17, miR-18a, miR-19a, miR-20a, miR-19b-1 *and *miR-92-1*) located on chromosome 13q31.3. The human genomic locus encoding these miRNAs undergoes amplification in several types of lymphoma and solid tumors. The *miR-17-92 *cluster seems to be tightly linked to the functions of the E2F family of transcription factors, which are critical regulators of the cell cycle and apoptosis. E2F1, E2F2, and E2F3 -activating E2Fs that induce expression of genes driving the progression from the G1 into the Sphase - were among the first experimentally verified targets of the *miR-17-92 *cluster. Moreover, both E2F1 and E2F3 can directly activate transcription of these miRNAs, thereby establishing a negative feedback loop [42]. The pro-tumorigenic activity of the *miR-17-92 *cluster also involves cell-nonautonomous functions that include induction of angiogenesis in solid tumors. Using a mouse model of colon cancer, Dews *et al*. demonstrated that the angiogenic activity of c-Myc is due at least in part to downstream activation of the *miR-17-92 *cluster. The anti-angiogenic factors thrombospondin-1 (TSP1) and connective tissue growth factor (CTGF) are negatively regulated by these miRNAs, which are potently induced by c-Myc in this model. Robust vascularization of tumors can be induced by expression of either c-Myc or the *miR-17-92 *cluster [43].

Overexpressed cyclooxygenase-2 (COX-2) strongly contributes to the growth and invasiveness of tumor cells in patients with CRC [44]. It has been demonstrated that COX-2 overexpression depends upon various cellular pathways involving both transcriptional and post-transcriptional regulations. An inverse correlation was reported between COX-2 and *miR-101 *expression in CRC cell lines. It was demonstrated *in vitro *that the direct translational inhibition of COX-2 mRNA is mediated by *miR-101*. Moreover, this correlation was supported by data collected *ex vivo*, in which colon cancer tissues and liver metastases derived from CRC patients were analyzed. Impairment of *miR-101 *levels could represent one of the leading causes of COX-2 overexpression in CRC cells [44].

### Polymorphisms within microRNA binding regions and risk of colorectal cancer

The binding of miRNA to mRNA is critical for regulating the mRNA level and protein expression. This binding can be affected, however, by single-nucleotide polymorphisms (SNPs) that can occur in the miRNA target site and can either abolish existing binding sites or create illegitimate binding sites. Therefore, SNPs within miRNA binding sites can have differing effects on gene and protein expression and represent another type of genetic variability that can influence the risk of certain human diseases, including CRC. Various approaches have been used to predict and identify functional SNPs within miRNA binding sites and their biological relevance is beginning to be evaluated in large case-control studies [45].

Regarding CRC, out of eight candidates predicted by computer simulation, the two genes for CD86, a costimulatory ligand on lymphocytes, and for the insulin receptor carry an SNP that are significantly associated with the risk of sporadic CRC (odds ratios 2.74 and 1.94, respectively) [46]. However, the biological relevance of these SNPs has not yet been confirmed by functional *in vitro *studies.

### MicroRNA expression in circulating blood as a new and promising early diagnostic option for colorectal cancer

Circulating nucleic acids (CNAs) offer unique opportunities for an early diagnosis of CRC. Dysregulated expression of miRNAs in various tissues has been associated with a variety of human cancers. More recently, miRNAs' occurrence in the serum and plasma of humans has been repeatedly observed. The levels of miRNAs in serum are more stable, reproducible, and consistent among individuals of the same species than are other CNAs [47,48]. The detection of serum miRNAs have been tested in prostate cancer, ovarian cancer and CRC patients as possible early diagnostic biomarkers [47-50].

In a study by Chen *et al*. [47], CRC patients had a significantly different serum miRNA profile compared to healthy subjects (HS). In all cases, 69 miRNAs were detected in the CRC serum but not in HS. It is of interest to note that CRC patients shared a large number of serum miRNAs (e.g., *miR-134, miR-146a, miR-221, miR-222, miR-23a*, etc.) with lung cancer patients. Pearson correlation further indicated that the levels of miRNAs in serum from lung cancer patients and CRC patients were consistent, suggesting that there are some "common" tumor-related miRNAs in serum [47]. Differentially expressed miRNAs in the plasma of patients with CRC have been also reported [50]. Expression pattern of 30 miRNAs (*miR-17-3p, miR-92, miR-135b, miR-222, miR-95*, etc.) in the plasma of patients with CRC were analyzed by real-time PCR expression profiling. Both *miR-17-3p *and *miR-92 *were significantly elevated (p < 0.0005). The plasma levels of these miRNAs were significantly reduced after surgery in 10 patients with CRC (p < 0.05) [50]. Further validation with an independent set of plasma samples (n = 180) indicated that miR-92 differentiates CRC not only from normal subjects but also from gastric cancer and inflammatory bowel disease. This marker yielded an ROC curve area of 88.5%. In discriminating CRC from control subjects, the sensitivity was 89% and the specificity was 70%. *miR-92 *has reasonable sensitivity for CRC detection and compares favorably with the fecal occult blood test [50].

### MicroRNA expression profiles of colorectal cancer tissue: implications for diagnostic oncology

Alterations in miRNA expression profiles have been successively detected in many types of human tumors [1,2]. The causes of the widespread differential expression of miRNA genes between malignant and normal cells can be explained by the gene location in cancer-associated regions, alterations in the miRNA processing machinery, and epigenetic mechanisms [1]. In reports on various cancer samples, generally lower miRNA levels were identified in tumors in comparison with normal tissue and, lower miRNA levels in poorly differentiated tumors compared to well-differentiated tumors in tissue samples [51] as well as in cell lines [52]. Studies focusing on miRNA expression profiling in CRC are summarized in Table 1, and some of these studies are described in detail below.

**Table 1:** Summary of studies focusing on miRNA expression profiling in colorectal cancer

Study	Design	Tumors (n)	Normal^1 ^(n)	Cell lines (n)	miR examined (n)	Technology	miR deregulated in tumors^2^
Michael *et al*. (53)	tumor vs. normal	14	4	2	28	Cloning	miR-143, miR-145, miR-21
Bandres *et al*. (54)	tumor vs. normal, TNM staging correlation	12	12	15	156	Real-time PCR	miR-31, miR-96, miR-133b, miR-135b, miR-145, miR-183
Cummins *et al*. (55)	miRNAs identification	4	2	5	x	miRAGE	133 novel microRNAs
Volinia *et al*. (56)	tumor vs. normal	46	8	0	245	miRNA microarray	miR-24-1, miR-29b-2, miR-20a, miR-10a, miR-32, miR-203, miR-106a, miR-17-5p, miR-30c, miR-223, miR-126, miR-128b, miR-21, miR-24-2, miR-99b prec, miR-155, miR-213, miR-150, miR-107, miR-191, miR-221, miR-9-3
Xi *et al*. (62)	tumor vs. normal, correlation with survival, p53 mutation status	24	24	0	10	Real-time PCR	miR-15b, miR-181b, miR-191, miR-200c
Lanza *et al*. (58)	MSS tumors vs. MSI tumors	23 MSS 16 MSI	0	0	230	miRNA microarray	comparison with colon epithelium was not analyzed
Slaby *et al*. (22)	tumor vs. normal, clinicopathological parameters correlation	29	6	0	5	Real-time PCR	miR-143, miR-145, miR-21, miR-31
Nakajima *et al*. (66)	tumor vs. normal, chemoresponse to S-1	21	21	0	5	Real-time PCR	let-7g, miR-181b, miR-200c
Monzo *et al*. (59)	tumor vs. normal and embryonic colon	22	22	0	156	Real-time PCR	Stage I (n = 28; miR-106a, miR-125b, miR-145, miR-17-5p, miR-200c, miR-21, miR-34a), Stage II (n = 64; miR-106a, miR-128a, miR-15a, miR-15b, miR-17-5p, miR-181a-c, let-7g, miR-200a-c, miR-21, miR-31, miR-34a, miR-92) *selection*
Schetter *et al*. (23)	tumor vs. normal, correlation with survival, TNM staging	84 (113)^3^	84 (113)^3^	0	389	miRNA microarray	miR-20a, miR-21, miR-106a, miR-181b, miR-203
Schepeler *et al*. (65)	tumor vs. normal, correlation with recurrence, MSS vs. MSI	49	19	0	315	miRNA microarray	miR-145, miR-455, miR-484, miR-101, miR-30b, miR-26b, miR-20a, miR-510, miR-92, miR-513, miR-526c, miR-320, miR-527, miR-432, miR-492, miR-200a, miR-191, miR-302a, miR-512-5p
Motoyama *et al*. (57)	tumor vs. normal, correlation with survival	4 (69)^3^	4 (69)^3^	0	455	miRNA microarray	miR-31, miR-183, miR-17-5p, miR-18a, miR-20a, miR-92, miR-143, miR-145
Ng *et al*. (50)	tumor, plasma vs. control	5 (25)^3^	5; (20)^3^	0	96	Real-time PCR	miR-18a, miR-223, miR-224, **miR-135b**, **miR-95**, miR-106a, miR-19a+b, **miR-17-3p**, miR-20a, **miR-92**, miR-221, miR-106b, **miR-222**, etc.; *markers upregulated in both plasma and tumors are given in bold type*

^1^Normal colon epithelium, ^2^only microRNAs deregulated in CRC in comparison to normal colon epithelium are mentioned, ^3^numbers of patients in the validation cohorts.

In 2003, Michael *et al*. [53] published the first such study. Using cloning technology followed by northern blotting, he observed consistently reduced accumulation of the specific mature *miR-143 *and *miR-145 *in the adenomatous and carcinoma stages of colorectal neoplasia. The same blots, however, displayed consistent levels of the ~70-bp *pre-miR-143 *in each of the cell lines. The authors concluded that the levels of mature *miR-143 *in these cells were controlled post-transcriptionally. These data suggested that abnormal processing might affect miRNAs expression in colon cancer cells [53].

Bandres *et al*. [54] examined by real-time PCR the expression of 156 mature miRNAs in colorectal tumors and adjacent non-neoplastic tissues from patients and CRC cell lines. This permitted them to identify a group of 13 miRNAs whose expression is significantly altered in this type of tumor. The most significantly deregulated miRNAs were *miR-31*, *miR-96*, *miR-135b*, *miR-183 (up-regulated in tumors and CRC cell-lines) and miR-133b, miR-145 (downregulated*). In addition, the expression level of miR-31 was positively correlated with the stage of CRC tumor [54]. These results, achieved through a standardized real-time PCR method, suggest that miRNA expression profile could have relevance to the biological and clinical behavior of colorectal neoplasia.

Velculescu's group developed an experimental approach called miRNA serial analysis of gene expression (miRAGE) and used it to perform one of the largest experimental analyses of human miRNAs [55]. Sequence analysis of 273,966 small RNA tags from human colorectal cells allowed them to identify 200 known mature miRNAs, 133 novel miRNA candidates, and 112 previously uncharacterized miRNA forms. To aid in evaluating the candidate miRNAs, they disrupted the Dicer locus in three human CRC cell lines and examined known and novel miRNAs in these cells. This study indicates that the human genome contains many more miRNAs than currently identified [55].

From a large-scale analysis of miRNA expression profiles on 540 samples of solid cancers, including CRC, Volinia *et al*. [56] identified a solid cancer miRNA signature composed by a large portion of overexpressed miRNAs. Among these miRNAs were some with well characterized cancer associations, such as miR-17-5p, miR-20a, miR-21, miR-92, miR-106a, and miR-155 [56]. A microarray-based approach for analysis of miRNA expression profiles in CRC was successfully applied also by Motoyama *et al*. [57].

In another profiling study, Lanza *et al*. [58] evaluated the expression of miRNAs and mRNAs in CRC samples characterized by microsatellite stability (MSS) or by high levels of microsatellite instability (MSI-H). Their analysis of miRNA expression profiles of MSI-H (*n *= 16) and MSS CRCs (*n *= 23) identified 14 differentially expressed miRNAs, while their analysis of messenger RNA expression profiles in these tumors identified 451 differentially expressed genes. Consequently, a smaller selected signature of best predictors of microsatellite status was generated: 27 genes, including 8 miRNAs, were identified as predictors. Further cluster analysis using just these 27 miRNAs and mRNAs also perfectly separated the two tumor classes. Cluster analyses run using either the mRNAs or the miRNAs independently did not perform as well in discriminating the tumor types. Therefore, the combined miRNA/mRNA fingerprint worked as the best discriminator for MSS versus MSI-H [58].

Monzo *et al*. [59] assessed the expression of mature miRNAs in human embryonic colon tissue, as well as in CRC and paired normal colon tissue. Overlapping miRNA expression was detected between embryonic colonic mucosa and CRC. The *miR-17-92 *cluster and its target, E2F1, exhibit a similar pattern of expression in human colon development and in colonic carcinogenesis - regulating cell proliferation in both cases [59]. Authors of this study conclude that miRNA pathways play a major role in both embryonic development and neoplastic transformation of the colonic epithelium.

From a diagnostic point of view, miRNA expression profiles might also contribute significantly to the further determination of the tissue origin of the cancer of unknown primary sites. Cancer of unknown primary (CUP) is usually a very aggressive disease with a poor prognosis. Identifying of colorectal cancer among adenocarcinoma of unknown primary site may improve prognosis of these patients by giving them a chance for modern anticancer targeted therapy. Two recent studies examined metastases of unknown primary tumors with miRNA microarrays for their potential to identify the tissue of origin. After establishing a miRNA classifier (n = 68, 11 tumor types, 217 miRNAs), 12 out of 17 poorly differentiated tumors were accurately classified by miRNA profiling [51]. A second publication reported an overall accuracy of 90% in classifying more than 400 malignant tumor samples of 22 tissue origins based on a set of 48 miRNAs [60]. A recent study on lymph node metastases of several malignant tumors, including CRC, identified three specific miRNAs (*miR-148a*, *miR-34b/c *and *miR-9*), specifically downregulated by CpG island hyper-methylation [61].

In general, there are several advantages of using miRNA expression profiling instead of its mRNA counterpart for biomarker identification and also for routine diagnostics. As a consequence of the fact that miRNAs target mRNAs with an imperfect sequence complementarity, a single miRNA can regulate the expression of more than 100 mRNAs simultaneously [4]. This might explain why microarrays of 217 miRNAs have much higher information content than 16,000 mRNAs in distinguishing different tissues and tumors [11]. It is relatively easier to discover reliable biomarkers from the approximately hundreds of miRNA candidates discovered to date than from over 40,000 genes. A further advantage is that, due to their small size and stem-loop structure, miRNAs are relatively more stable and less subjected to degradation during fixation and sample processing. One recent examination compared the miRNA-expression profiles from fresh frozen versus formalin-fixed paraffin-embedded (FFPE) CRC tissues. A good correlation coefficient of 0.86-0.89 was observed. Worthy of note is that differing formalin fixation times - inevitable in a routine pathology lab - did not significantly influence the expression of miRNAs in 40 CRC specimens [62]. This can also be of benefit for large retrospective studies based on archived FFPE samples. Furthermore, miRNAs can be visualized at the cellular and sub-cellular levels by conventional as well as fluorescence *in situ *hybridization [63].

### Potential of microRNA expression in cancer prognosis and prediction

**Figure 2:**
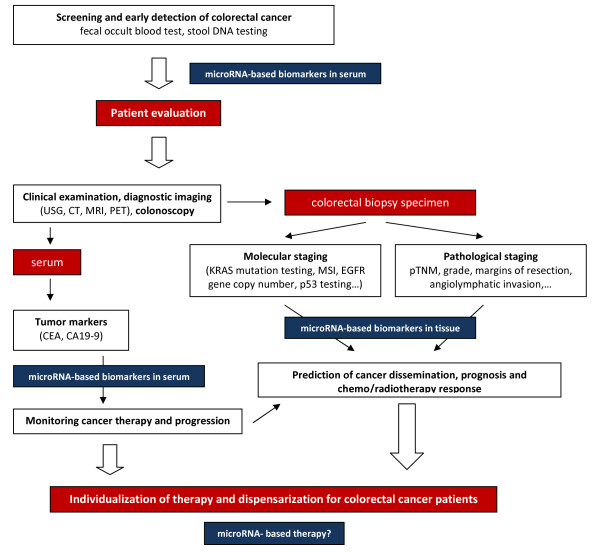
**The potential usage of miRNAs in the clinical management of the colorectal cancer patients**.

Accumulating evidence shows that miRNA expression patterns are unique to certain cancers and have potential to be used as prognostic and predictive factors in clinical routine (see Fig. 2). Xi *et al*. performed Kaplan-Meier analysis for CRC patients with International Union Against Cancer (UICC) stages I-IV and found that tumors expressing high levels of *miR-200c*, recently connected to EMT, are correlated with poorer prognosis, regardless of tumor stage [64]. These investigators also found that p53 mutation, commonly found in CRC, is strongly associated with greater than twofold *miR-200c *overexpression.

*miR-21 *is upregulated in many solid tumors, including CRC [21]. Recently, we have found that *miR-21 *overexpression shows a strong correlation with the established prognostic factors as nodal stage, metastatic disease and UICC stage [22]. Using class comparison analysis, Shetter *et al*. [23] later found that 37 miRNAs were differentially expressed in tumors of CRC patients. From this group, *miR-20a*, *miR-21*, *miR-106a*, *miR-181b*, and *miR-203 *were found by Cox regression analysis to be associated also with poor survival and were selected for validation. Validation was performed by measuring miRNAs' expression using real-time PCR in tumor and paired non-tumor tissues in the validation cohort [23]. In the validation set, only high expression of *miR-21 *was significantly associated with poor prognosis, and this association was independent of age, sex, and tumor location. Multivariate analysis further revealed that high *miR-21 *expression in tumors was associated with poor survival, independent of the tumor stage. In patients who received adjuvant therapy, high *miR-21 *expression indicated a poor response to therapy [23].

Schepeler *et al*. [65] found that miRNAs were associated with tumor microsatellite status in stage II colon cancer. The predictive molecular signature was composed of only four miRNAs (*miR-142-3p*, *miR-212*, *miR-151*, and *miR-144*). Furthermore, a biomarker based on miRNA expression profiles could predict recurrence of disease with an overall performance accuracy of 81%, thus, indicating a potential role for miRNAs in determining tumor aggressiveness. Kaplan-Meier survival curves showed that patients who had stage II CRC tumors with high expression of *miR-320 *or *miR-498 *had significantly shorter progression-free survival than did patients whose tumors showed low expression. These miRNAs were correlated with the probability of progression-free survival also by multivariate analysis [65]. Although these results are promising, larger studies will be needed to prove whether miRNAs really have significant potential to extend prognostic information based on the recent standard diagnostic procedures.

Another important question for management of CRC patients is the possibility of predicting therapy response. Nakajima *et al*. [66] evaluated the significance of five mature miRNAs in tumors of CRC patients treated with 5-fluorouracil-based antimetabolite S-1. They identified *let-7g *and *miR-181b *as significant indicators for chemoresponse to S-1-based chemotherapy. A study published by Rossi *et al*. [67] reported a suggestive pattern of miRNAs rearrangement in HT-29 and HCT-116 human colon cancer cell lines after exposure to 5-fluorouracil (5-FU), a classical antimetabolite in broad clinical use. At a clinically relevant concentrations, the drug upregulated or downregulated *in vitro *the expression of 19 and 3 miRNAs, respectively, by a factor of not less than two-fold. In some instances, 5-FU upregulated miRNAs that are already overexpressed in tumor tissue, including, for example, *miR-21 *[67]. In other instances, by contrast, the drug influenced the expression of miRNAs in a direction that is opposite to that induced by neoplastic transformation. A typical example is provided by *miR-200b*, which is upregulated in various tumors but downregulated by treatment with 5-FU. Interestingly, it is known that *miR-200b *targets mRNA that codes for a protein tyrosine phosphatase (PTPN12) which inactivates products of oncogenes, such as ABL, SRC or KRAS [65]. Another study evaluated changes in miRNA expression profiles as a response to therapy, focusing on the effects of capecitabine chemoradiotherapy on rectal tumors *in vivo *[68]. Tumor microexcisions were taken before starting a therapy and, again, after a two-week therapy. The extent of tumor response to the therapy was investigated microscopically by an experienced pathologist according to Mandard's tumor regression criteria. In this study, many miRNAs (*miR-10a*, *miR-21*, *miR-145*, *miR-212*, *miR-339*, *miR-361*) responded to capecitabine chemoradiotherapy in individual tumor samples. In most samples, however, only two miRNAs, *miR-125b *and *miR-137*, showed significant increase in expression levels after two-week therapy [68]. Despite these results, more studies are needed that will examine the effects of chemotherapeutic agents on the miRNA expression profiles and their possible usage for predicting therapy response in CRC patients.

### Silencing and recovery of altered microRNAs - a future therapeutic approach in colorectal cancer?

Since miRNAs constitute a robust network for gene regulation, they possess a great potential as both, a novel class of therapeutic targets and a powerful intervention tool. The biosynthesis, maturation and activity of miRNAs can be manipulated with various oligonucleotides that encode the sequences complementary to mature miRNAs [12]. Overexpression of miRNAs can be induced either by using synthetic miRNA mimics or chemically modified oligonucleotides. Conversely, miRNAs can be silenced by antisense oligonucleotides and "antagomirs" (synthetic analogues of miRNAs). Cross-sensitivity with endogenous miRNAs and lack of specificity for cancer cells can cause nonspecific side effects during miRNA modulation therapy. However, the use of an effective delivery system and less toxic synthetic anti-miRNA oligonucleotides may minimize such side effects [69,70]. Gene therapies may be designed to treat CRC and to block the progression of precursor lesions by manipulating the tumor suppressive or oncogenic miRNAs. Such manipulation may control the tumor growth rate and have potential as a new therapy for both early and advanced cancers [71].

Studies have revealed that inhibition of *miR-21 *and *miR-17-92 *activity is associated with reduced tumor growth, invasion, angiogenesis and metastasis [21,43]. Moreover, overexpression of *miR-21 *is associated with low sensitivity and a poor response to chemotherapy, and its inhibition may improve the response to chemotherapy [21]. On the other hand, restoration of *miR-145 *expression has been associated with inhibition of tumor cells growth via downregulation of IRS-1. Expression levels of *miR-145*, downregulated in tumor tissues of CRC patients, were increased *in vitro *and caused reduced cell proliferation and increased sensitivity to radiotherapy [41]. These miRNAs present only examples of miRNAs validated as oncogenes or tumor suppressors in CRC and thus of potential candidates for miRNA-based targeted CRC therapy. Targeting such miRNAs may help to not only prevent the recurrence of disease in high-risk tumors in UICC stage II and control the growth of advanced metastatic tumors, but they also could provide another possibility for chemo- and radio-resistant cancer patients. Although experimental miRNA therapy results look promising, only a limited number of studies have been conducted under *in vivo *conditions in animal models. There is still a long way to go to reach clinical testing of the first miRNA-based therapy for CRC in the future.

## Conclusion

The discovery of miRNAs has substantially changed the view on gene regulation, and new findings over the past few years have catapulted miRNAs to the center stage of cancer molecular biology. It is now evident that dysregulation of miRNAs is an important step in the development of many cancers, including CRC. A number of studies based on expression profiling has proven there are significant changes of miRNA expression levels in CRC tissue in comparison to colorectal epithelium, and these have identified groups of miRNAs enabling prognostic stratification of CRC patients and prediction of their responses to selected chemotherapeutic regimens and radiotherapy. To improve knowledge as to the roles of miRNAs in CRC pathogenetic pathways, functional effects of particular miRNAs have been successfully studied. The results of these studies suggest great potential for miRNAs as a novel class of therapeutic targets and as a powerful intervention tool in CRC. miRNAs' occurrence has been repeatedly observed also in serum and plasma, and miRNAs as novel minimally invasive biomarkers have indicated reasonable sensitivity for CRC detection and compare favorably with the fecal occult blood test. The potential use of miRNAs in the clinical management of CRC patients is summarized in Figure 2.

## Competing interests

The authors declare that they have no competing interests.

## Authors' contributions

OS and MS drafted and wrote the manuscript. JM and RV revised the manuscript critically for important and intellectual content. All authors read and approved the final manuscript.
